# Alternative Splicing of the Last *TKFC* Intron Yields Transcripts Differentially Expressed in Human Tissues That Code In Vitro for a Protein Devoid of Triokinase and FMN Cyclase Activity

**DOI:** 10.3390/biom14101288

**Published:** 2024-10-12

**Authors:** María Jesús Costas, Ana Couto, Alicia Cabezas, Rosa María Pinto, João Meireles Ribeiro, José Carlos Cameselle

**Affiliations:** Departamento de Bioquímica y Biología Molecular y Genética, Facultad de Medicina, Universidad de Extremadura, 06006 Badajoz, Spain; macostas@unex.es (M.J.C.); anacouto0406@gmail.com (A.C.); acabezas@unex.es (A.C.); pincor@gmail.com (R.M.P.)

**Keywords:** triokinase, FMN cyclase, human *TKFC* gene, alternative splicing

## Abstract

The 18-exon human *TKFC* gene codes for dual-activity triokinase and FMN cyclase (TKFC) in an ORF, spanning from exon 2 to exon 18. In addition to TKFC-coding transcripts (classified as *tkfc* type by their intron-17 splice), databases contain evidence for alternative *TKFC* transcripts, but none of them has been expressed, studied, and reported in the literature. A novel full-ORF transcript was cloned from brain cDNA and sequenced (accession no. DQ344550). It results from an alternative 3′ splice-site in intron 17. The cloned cDNA contains an ORF also spanning from exon 2 to exon 18 of the *TKFC* gene but with a 56-nt insertion between exons 17 and 18 (classified as *tkfc_ins56* type). This insertion introduces an in-frame stop, and the resulting ORF codes for a shorter TKFC variant, which, after expression, is enzymatically inactive. *TKFC* intron-17 splicing was found to be differentially expressed in human tissues. In a multiple-tissue northern blot using oligonucleotide probes, the liver showed a strong expression of the *tkfc*-like splice of intron 17, and the heart preferentially expressed the *tkfc_ins56*-like splice. Through a comparison to global expression data from massive-expression studies of human tissues, it was inferred that the intestine preferentially expresses *TKFC* transcripts that contain neither of those splices. An analysis of transcript levels quantified by RNA-Seq in the GTEX database revealed an exception to this picture due to the occurrence of a non-coding short transcript with a *tkfc*-like splice. Altogether, the results support the occurrence of potentially relevant transcript variants of the *TKFC* gene, differentially expressed in human tissues. (This work is dedicated in memoriam to Professor Antonio Sillero, 1938–2024, for his lifelong mentoring and his pioneering work on triokinase).

## 1. Introduction

The human *TKFC* gene (GeneID 26007, Ensembl ID ENSG00000149476) codes for the dual-activity enzyme named triokinase (EC 2.7.1.28) and FMN cyclase (EC 4.6.1.15) [1,2]. The triokinase activity catalyzes the third step of the specific metabolism of fructose (ATP-dependent phosphorylation of D-glyceraldehyde) [3,4,5,6] and the phosphorylation of the exogenous genotoxic dihydroxyacetone [7,8,9,10]. The enzyme also exerts control on the lipogenic potential of fructose and dietary tolerance [11]. The FMN cyclase activity catalyzes the cyclizing lyase splitting of FAD to AMP and cFMN (riboflavin 4′,5′-cyclic phosphate) [12,13]. This unusual flavin nucleotide occurs in nanomolar concentrations in rat livers with an unknown function [14], and it is an abundant component of the posterior flagellum of reproductive cells in the brown alga *Scytosiphon lomentaria*, where it could be involved in phototaxis [15]. In addition to its enzymatic activities, the TKFC protein interacts with melanoma differentiation antigen 5 (MDA5), an RNA helicase acting as a viral RNA sensor during viral replication, and as an inducer of interferon expression. Through this interaction, TKFC inhibits MDA5-mediated innate antiviral signaling [16,17]; interestingly, this effect can be modulated via the binding of non-coding RNAs (microRNA-122 or circSOBP) to TKFC [18,19]. Additionally, the *TKFC* promoter is controlled by the carbohydrate response element-binding protein (ChREBP) [20,21,22].

**Scheme 1:**
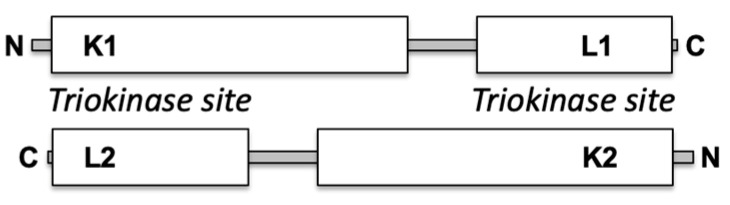
Domains in the subunits of homodimeric TKFC with two triokinase active sites.

TKFC is a homodimeric protein of two-domain monomers, each containing a K domain (N-terminal) and an L domain (C-terminal). In the homodimer, two triokinase active sites are formed between the K1–L2 and the K2–L1 domains (Scheme 1). In contrast, the FMN cyclase activity requires only the L domain, although full cyclase efficiency also needs the K domain [2]. Interaction of TKFC with MDA5 occurs through the K domain, which, when tested in isolation, is an effective inhibitor of MDA5-mediated innate immunity comparable to the effect of the full TKFC protein [16].

Human *TKFC* bi-allelic mutations that inactivate the triokinase activity are present with different phenotypes depending on the protein domain where the altered residue(s) are located. There is a report of an individual with compound heterozygous missense mutations in the K domain (c.574G>C (p.Gly192Arg) and c.682C>T (p.Arg228Trp)), which presents an isolated hair phenotype (hypotrichosis with loose anagen hairs) [23]. On the other hand, several individuals with homozygous variants in the L domain have been reported to display more severe phenotypes. The presence of either c.1628G>T (p.Arg543Ile) or c.1333G>A (p.Gly445Ser), both in homozygosis, have been linked to cataracts and multisystem diseases, including developmental delay, liver dysfunction, microcytic anemia, and fatal cardiomyopathy [24]. Recently, the presence of the homozygous c.1624G>A (p.Gly542Arg) variant has been shown, in one individual, to be associated with a complex primary immunodeficiency in isolation [25].

Very recently, the homozygous c.598G>A p.(Val200Ile) variant of *TKFC* gene was reported in two neonates with skeletal abnormalities and fatal hypertrophic cardiomyopathy [26]. The relationship of this variant to altered TKFC activities is compounded because no enzyme activity data are available, and the patients also display homozygous variants in the *TYR* gene.

**Figure 1:**
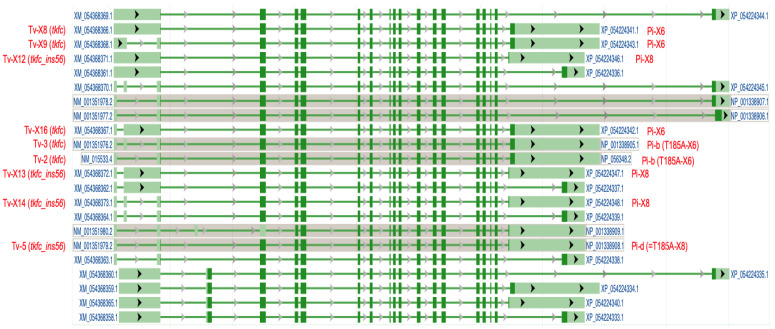
Exon-intron structure of human full-length *TKFC* transcript variants as shown in the NCBI Genome Data Viewer (chromosome 11, alternate T2T-CHM13v2.0 genome). Dark green colored regions indicate the coding sequences. The transcript variants labeled in red type are those that code for fully active TKFC protein (isoforms b and X6) or for the inactive isoform cloned and characterized in this manuscript (isoforms d and X8). See also Table 1. Tv, transcript variant; Pi, protein isoform.

According to the mRNA NM_015533.4 (which is the MANE Select transcript for the human *TKFC* gene [27]) aligned to genomic DNA (chromosome 11q12.2), the *TKFC* gene (ID: 26007) is depicted as containing 18 exons. The ORF coding for TKFC protein (1728 nt; accession no. ABA10576.1) starts in the 3′ end of exon 2 and ends within exon 18, giving rise to a 575 amino acid translation, which, once expressed in homodimeric form, displays triokinase and FMN cyclase activities [1,2,28]. This ORF is present in five transcript variants with different splicing patterns which code for the protein isoform b or X6 (they differ by a single amino acid in position 185 due to a common SNP [28]) (Table 1 and Figure 1). Current evidence indicates that one or more of these transcripts and the protein isoform they encode are the major products of the human *TKFC* gene. However, as summarized in Table 1 and Figure 1, there is evidence for alternative spliced full-length transcript variants which code for different protein isoforms, although none of them has been expressed and studied so far. Here, we report on the cloning, expression, and characterization of the ORF encoded by transcript variants X12–X14 and their product protein isoform X8.

## 2. Materials and Methods

### 2.1. PCR Cloning, Construction, and Sequencing of Plasmid pGEX-6P-3-hF1

The ORF for human TKFC isoform X8 was amplified with Pfu-Ultra High-Fidelity DNA polymerase (Stratagene) from brain cDNA (Marathon Ready cDNA; BD Biosciences). The design of the primers was based on the sequence of transcript NM_015533 which codes for TKFC isoform b. The forward primer was CATCTGTCGACATGACCTCCAAGAAGCTG, which includes a SalI site (underlined) followed by the first 18 nucleotides of the ORF of isoform b. The reverse primer was TTACAGCGGCCGCCTAGCTCTGCAAGACCTC, which includes a NotI site (underlined) followed by the reverse complement of the 18 last nucleotides of the ORF. An amplicon near the expected size was obtained and inserted in the pGEX-6P-3 plasmid (Amersham) between the SalI and NotI sites. Twelve clones containing an insert were isolated and sequenced. Eleven plasmids contained the 1728 bp ORF coding for TKFC isoform X6 [1], and only one contained the 1784 bp ORF coding for TKFC isoform X8. Plasmid pGEX-6P-3-hF1 was submitted to double-strand sequencing and used for expression. The results of this manuscript were previously undisclosed except in the deposit in GenBank (accession no. DQ344550) on 24 January 2006 by our laboratory.

### 2.2. Expression of Plasmid pGEX-6P-3-hF1 in BL21 Cells

pGEX-6P-3-hF1 was used to transform BL21 cells, which were seeded in LB agar plates with 0.1 mg/mL ampicillin. One clone was picked and grown overnight at 37 °C in 10 mL of liquid LB medium with ampicillin (amp-LB). Five milliliters were inoculated in 100 mL of amp-LB incubated at 27 °C with shaking. When *A*_600_ reached 0.6–0.8, 0.5 mM IPTG was added. After 2 h at 27 °C, the cells were pelleted by 10 min centrifugation at 9000 rpm at 4 °C in a Sorvall SS34 rotor. The cell pellet was resuspended in 10 mL of 20 mM Tris-HCl, pH 7.5, 0.5 mM EDTA, 1 mM DTT, 50 mM KCl, containing one tablet of Complete^TM^ protease inhibitor cocktail (Mini, EDTA-free; from Roche). Sonication on ice followed at 170 W for 5 min (0.1 s pulses per second) with three repetitions. The lysate was centrifuged at 15,000 rpm for 10 min at 4 °C in a Sorvall SS34 rotor. The supernatant was used to purify the GST-X8 fusion protein. The precipitate was resuspended in 10 mL of sonication buffer for analytical purposes.

### 2.3. Purification of TKFC Isoform X8

Ten milliliters of the supernatant containing the GST-X8 fusion protein was applied to a 1.5 mL glutathione-Sepharose column equilibrated in 20 mM Tris-HCl, pH 7.5, 0.5 mM EDTA, 1 mM DTT, and 50 mM KCl. The column was washed with 15 mL of 50 mM Tris-HCl, pH 7.5, 1 mM EDTA, 1 mM DTT, and 150 mM NaCl to eliminate the unbound protein. After this, 20 µL of PreScissionTM protease (Amersham), diluted in 1.5 mL of wash buffer, was applied to the column, and flow was stopped. After 12 h at 4 °C, the product of in-column specific proteolysis was recovered with 4.5 mL of wash buffer. The fractions collected during and after protease treatment were analyzed by SDS-PAGE.

### 2.4. Triokinase and FMN Cyclase Assays of Recombinant TKFC Isoform X8

The absence of enzyme activity of isoform X8 was tested using the assay methods described earlier. In brief, triokinase activity was tested with dihydroxyacetone and ATP as substrates using a continuous spectrophotometric assay coupled to glycerol 3-phosphate dehydrogenase as indicator enzyme [2]. The FMN cyclase or FAD-AMP cyclizing lyase activity (FAD → cFMN + AMP) was tested discontinuously by colorimetric assay of the liberation of inorganic phosphate from AMP by alkaline phosphatase, used as a coupling enzyme [13].

### 2.5. Multiple Tissue Northern Blot

A commercial northern membrane containing twelve lanes with 1 µg poly(A) RNA per lane from different human tissues was purchased from BD Biosciences, Clontech (catalog no. 7780-1). For hybridization, two 50-mer oligonucleotides were designed: E17E18 (TGGCCTCGGCTGCAGCTTCGGCACTCTTGACTGCTTTGGTCAGGACTTGT) composed by the reverse complement of the 5′-most 25 nt of *TKFC* exon 18 (underlined) followed by the reverse complement of the 3′-most 25 nt of *TKFC* exon 17; INS50 (GATGGGGTGTGGGAGCAGGTTCAAGGGCAGATCACCAGGCCGCCTCCTTC), which is part of the reverse complement of the 56-nt insertion left by the functioning of the alternative 3′-splice site of *TKFC* intron 17. The probes, E17E18 and INS50, were labeled with digoxigenin-dUTP using the DIG Oligonucleotide Tailing Kit, 2nd Generation (Roche), according to the manufacturer’s instructions. Hybridizations (2 h) were performed essentially as reported [29], except that the (pre)hybridization solutions were supplemented with 50 mg/L polyA and used, like the washing buffer, at 45 °C. Chemiluminescence was recorded by 120 min exposures of X-ray film to the hybridized membrane. First, the membrane was processed with the E17E18 probe. Thereafter, the blot was stripped and reprobed with INS50.

### 2.6. Molecular Modeling

The most recent molecular model of wild-type homodimeric TKFC, which corresponds to protein isoform X6, has been described by Onoufriadis et al.; it was validated using a combination of homology modeling and molecular dynamics simulation [23]. Using this model of isoform X6, the 50 amino acids lost due the alternative splicing on intron 17 were highlighted by showing them in a different color, or just by hiding them. These digital manipulations were conducted with VMD v.1.9.4a57 software [30].

## 3. Results

### 3.1. Cloning and Sequence Analysis of the Coding Sequence of TKFC Protein Isoform X8 Formed by Intervention of an Alternative 3′-Splice Site in TKFC Intron 17

**Figure 2:**
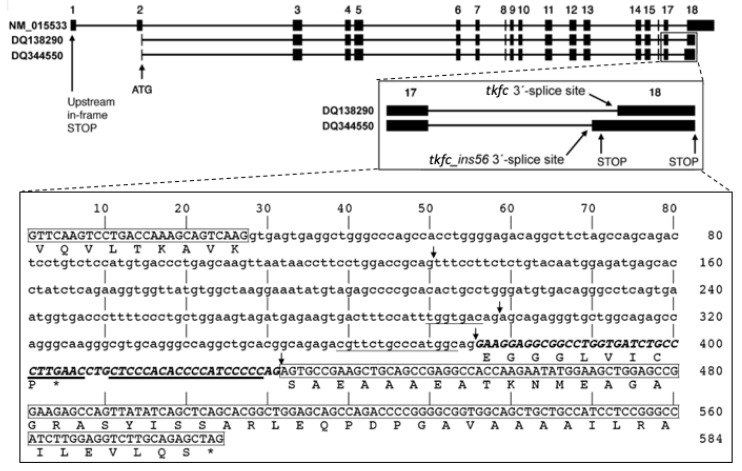
Exon-intron structure of the human *TKFC* gene and alternative transcripts produced by the use of different 3′-splice sites in intron 17. NM_015533 is the MANE Select [27] spliced transcript of the *TKFC* gene used as reference to define the gene structure. DQ138290 is the spliced ORF that encode the 575 amino-acid protein isoform X6 which is active as triokinase and FMN cyclase [1,2,28]. DQ344550 is the novel *tkfc_ins56*-type spliced sequence reported here, which codes for protein isoform X8. The inserts are enlargements showing the alternative splicing of *TKFC* intron 17 and the locations of stop codons. The larger insert shows the sequence of intron 17 flanked by exon sequences. Boxed sequences are part of exon 17 (3′-end) and exon 18 (5′-end). The non-boxed part of the sequence corresponds to full intron 17 according to the *tkfc*-type splicing. The uppercase sequence within intron 17 is the 56 nt insertion (*ins56*) that remains in the alternative *tkfc_ins56*-type splicing. The underlined sequences mark the possible branching sites and the polypyrimidine tracts near each 3′-splice site. The arrows mark the acceptor sites identified by the neural network predictor program NNSPLICE 0.9 with scores higher than 0.05 for a 0–1 range: 0.22 (at 130-1), 0.62 (at 298-9), 0.21 (at 375-6; the site used to form *tkfc_ins56*-type transcripts) and 0.39 (431-2; the site used to form *tkfc*-type transcripts) [31]. The amino acid translation shown below exon 17 is common to protein isoform X6 and X8. The translation shown below the 56 nt insertion, ending with a proline residue, is the C-terminal sequence unique to protein isoform X8 (accession no. ABC70184), and that below exon 18, ending with a serine residue, is unique to the catalytically active isoform X6 (accession no. ABA10576). Stop codons are indicated by asterisks.

The PCR cloning of human brain TKFC was performed using primers designed to amplify the 1728 nt ORF, from the ATG to the stop codon, based on the MANE Select mRNA NM_015533 [1]. Eleven out of the 12 pGEX-6P-3 clones then sequenced were derived from transcripts constitutively spliced in intron 17 (i.e., they are of *tkfc* type). The remaining plasmid clone, named pGEX-6P-3-hF1, contained a novel sequence of 1784 nt (GenBank accession no DQ344550) that fits with transcripts alternatively spliced in intron 17, leaving a 56-nt insertion between exons 17 and 18 (i.e., they are of *tkfc_ins56* type). The 1784 nt amplicon contained in pGEX-6P-3-hF1 was like the 1728 nt one, except for a silent G/A SNP at position 507 and for a 56 nt insertion (*ins56*) just after position 1575, i.e., in the junction of *TKFC* exons 17-18 (Figure 2). The sequence of *ins56* aligned exactly with the 3′ end of intron 17 in the genomic *TKFC* sequence and delimited an alternative 3′-splice site with a weak consensus motif: it had the invariant AG, but its polypyrimidine tract contained several purines, and the nearest fit to the branching point consensus was at a long distance, i.e., 80-nt away from the alternative intron end (Figure 2, larger insert). The functionality of the alternative 3′-splice site denoted by the *tkfc_ins56*-type splice was confirmed by the neural network predictor NNSPLICE 0.9 [31], which found it as one of four possible 3′-splice sites within intron 17 (Figure 2, larger insert, arrows).

Compared to transcripts that code for fully active TKFC (isoforms b or X6), the insertion of *ins56* in the junction of exons 17–18 led to a shorter protein isoform, as the inserted sequence contained an in-frame stop codon. While the ORF of transcripts coding for isoforms b or X6 yielded the 575 amino-acid TKFC [1,2,28], after the 56 nt insertion, the resulting ORF translated to a shorter, 534 amino-acid protein (isoform X8), with the peptide EGGGLVICP substituting for the 50 C-terminal residues of TKFC (Figure 2, larger insert). An interesting feature of this premature termination is that it occurs within the last exon of the transcripts, so that they can escape mRNA degradation via the nonsense-mediated decay pathway, which, in mammals, is related to premature stop signals at least 50–55 nt upstream of the last exon-exon junction [32].

### 3.2. Expression and Catalytic Inactivity of the Recombinant Protein Isoform X8

**Figure 3:**
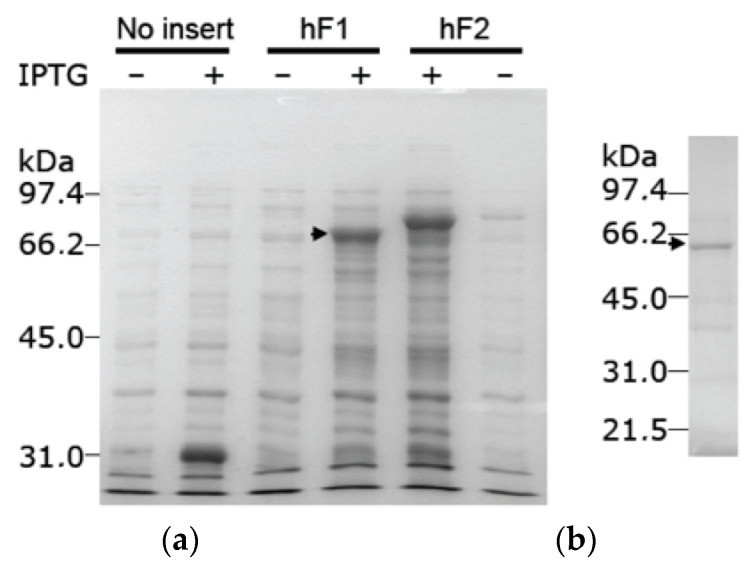
Expression of the recombinant protein isoform X8, encoded by an alternatively spliced *tkfc_ins56*-type transcript. (**a**) IPTG-dependent expression of GST fusion proteins in supernatant lysates of *E. coli* BL21 cells transformed with plasmids pGEX-6P-3 (without insert), pGEX-6P-3-hF1 (bearing the *tkfc_ins56*-type ORF) or pGEX-6P-3-hF2 (bearing the *tkfc*-type ORF [1]). Inserts were cloned in frame with the GST tag. (**b**) Protein recovered from the GST-tagged expression product after adsorption to GSH-Sepharose and overnight in-column proteolysis with PreScission^®^ protease. The arrowheads mark the GST fusion proteins with isoform X8 (**a**) or the protein separated from the GST tag (**b**). Original images of (**a**,**b**) can be found in Supplementary Materials.

The protein isoform X8 was expressed as a GST fusion protein upon IPTG induction of *E. coli* BL21 cells transformed with plasmid pGEX-6P-3-hF1. The molecular weight of the induced protein was, as expected, somewhat smaller than the corresponding fusion protein formed with active TKFC (Figure 3a). The GST-tagged protein was adsorbed to a GSH-Sepharose column and was recovered by in-column specific proteolysis that separated it from the GST tag (Figure 3b). In these preparations, the enzyme activities typical of triokinase and FMN cyclase were undetectable, i.e., below 3% of the specific activities measured with active enzyme expressed and prepared in parallel with the same system (Figure 3a).

**Figure 4:**
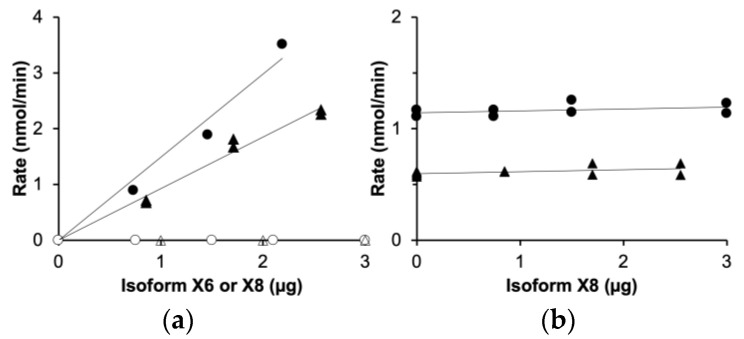
Inactivity of TKFC isoform X8 compared to isoform X6: assays of dihydroxyacetone kinase (circles) and FMN cyclase (triangles). (**a**) Assays run with either isoform X6 (filled symbols) or isoform X8 (empty symbols). (**b**) Assays run with a constant amount of isoform X6 (0.73 µg) and variable concentrations of isoform X8.

In the purified isoform X8 (Figure 3b), the enzyme activities typical of triokinase and FMN cyclase were undetectable, below 3% of the rates measured with the active isoform X6. This was shown by assays of the individual isoforms X6 and X8 (Figure 4a) and of X6/X8 mixtures in different proportions (Figure 4b), clearly demonstrating the inactivity of isoform X8.

**Figure 5:**
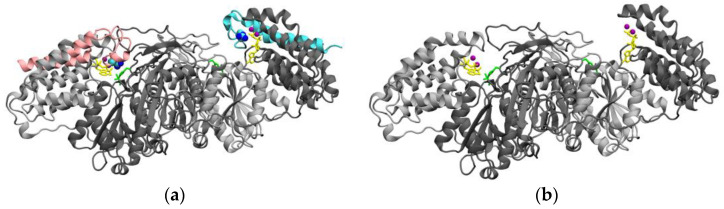
Comparison of the structure of active TKFC protein (isoform X6) with the catalytically inactive isoform X8 studied in this manuscript. (**a**) Homodimeric structure of TKFC isoform X6 [1,2,28]. The two subunits are shown in different gray tones, except that the 50 C-terminal amino acids encoded by exon 18 (Figure 2, large insert) are colored either pink or blue in each subunit. The essential Arg543 residues (blue spheres) that interact with ATP (yellow) are highlighted. (**b**) Parts of isoform X6 that remain in isoform X8. The model is like that of part (**a**), except that the 50 C-terminal amino acids of each subunit have been removed, showing the disappearing of Arg543 residues. To fully represent the structure of isoform X8, the peptide EGGGLVICP encoded by intron 17 should be added (Figure 2, large insert). Anyhow, since isoform X8 is enzymatically inactive, it is clear that this peptide cannot play the role of Arg543.

The inactivity of isoform X8 is related to the absence of the 50 C-terminal amino acids of the active enzyme (isoform X6). According to the most recent structural model of active TKFC [23], the missing amino acids are part of the L domain, which is required for the FMN cyclase activity [2] and includes a 20 amino-acid loop (residues 535–554) that contains Arg-543 which is essential for triokinase activity [23] (Figure 5a,b), just as it is the conserved residue in the *Escherichia coli* homolog [33].

### 3.3. Tissue Expression of TKFC-Gene Transcripts Formed with Alternative 3′-Splice Sites of Intron 17 Studied by Northern Analysis

**Figure 6:**
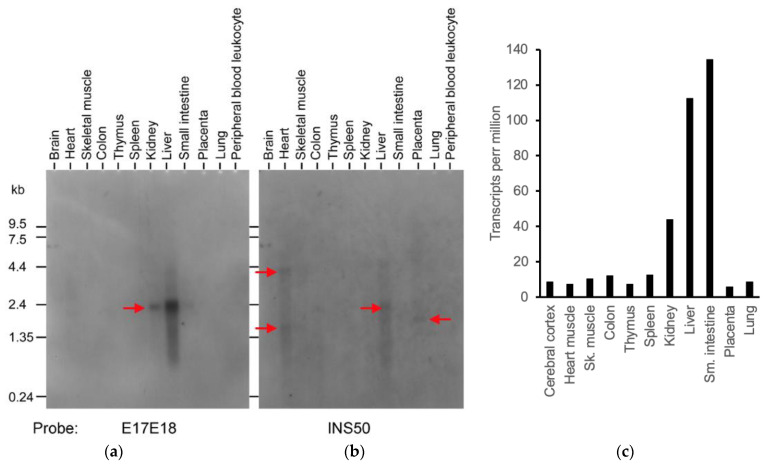
Differential expression of the alternative splices of *TKFC* intron 17 in several human tissues. (**a**) Expression of the *tkfc*-like splice containing an exon 17-18 junction. (**b**) Expression of the *tkfc_ins56*-like splice containing an insertion between exons 17 and 18. The red arrows mark the major RNAs detected. (**c**) Total expression of the *TKFC* gene in the human tissues as determined by RNA-Seq (https://www.proteinatlas.org/ENSG00000149476-TKFC/tissue, accessed on 25 August 2024; RNA expression, Consensus dataset of the Human Protein Atlas; accessed on 10 June 2024) [34]. Similar data were found in the Genotype-Tissue Expression (GTEx) database [35] (https://gtexportal.org/home/gene/TKFC; accessed on 25 August 2024). Original images of (**a**,**b**) can be found in Supplementary Materials.

To estimate the relative levels of human transcripts processed at *TKFC* intron 17, like *tkfc*- or *tkfc_ins56*-type transcripts, a Multiple Tissue Northern (MTN) blot was probed with two 50-mer oligonucleotides one after another: E17E18 and INS50 (Figure 6a,b). According to BLASTN searches run against human genomic sequences, these oligonucleotides were uniquely related to the *TKFC* gene. The probes were, respectively, designed to hybridize with transcripts containing the exon 17-18 junction like the *tkfc*-type, or with the 56 nt insertion typical of *tkfc_ins56*-type transcripts. The E17E18 and INS50 probes disclosed different patterns of expression and, interestingly, they did not correlate with the global expression profile of the *TKFC* gene according to databases [34,35] (Figure 6c).

Concerning the expression patterns inferred from the Northern blot, hybridization with E17E18 revealed a 2.2 kb transcript strongly expressed in the liver and less so in kidney, spleen, and small intestine (Figure 6a). Reprobing with INS50 gave no strong hybridization signal, but weak ones were observed in the heart (4.3 kb and 1.6 kb), liver (2.2 kb), and placenta (1.8 kb) (Figure 6b). One aspect worthy of comment was the absence of clear E17E18 and INS50 signals in brain tissue, despite the fact that the cDNA clones considered in this and previous work were amplified by PCR from a human brain cDNA library (Section 2.1). This may be explained by an insufficient sensitivity of the northern assay for the relatively low abundance transcripts captured by PCR cloning. Nonetheless, the sensitivity of the northern blot was clearly enough to make conclusions concerning different patterns and relative levels of expression. Particularly noteworthy was the difference between the heart and liver samples. In addition to the different size of the predominant transcripts in each source, the detection of INS50 but not E17E18 signals in the heart makes a strong case for the preferential in vivo use of the ‘alternative’ 3′-splice site of *TKFC* intron 17, which is contrary to the situation in the liver.

### 3.4. Tissue Expression of TKFC-Gene Transcripts Quantified by RNA-Seq

Tissue expression data for individual transcript variants, quantified from RNA-Seq experiments using the RSEM software (RNA-Seq by Expectation Maximization [36]), can be found in the GTEx database [35]. Concerning the *TKFC* gene, besides global expression data from 54 human tissue locations, individual data for 21 Ensembl transcript variants were recorded (https://gtexportal.org/home/gene/TKFC, accessed on 25 August 2024). Out of these, only two transcript variants are clearly related to those deposited in GenBank (Figure 1) and to the two cDNAs from where protein isoforms X6 [1,2,28] and X8 (this work) have been expressed. ENST00000394900.7 is a full-length 4,678 nt transcript, 100% identical to GenBank sequence NM_015533.4 (Tv-2 in Figure 1), which codes for enzymatically active TKFC (protein isoform b or T185A-X6). ENST00000529479.5 is a 1781 nt transcript identical to GenBank sequence DQ344550 (which codes for the inactive TKFC isoform X8), except for three SNPs (with two amino acid substitutions) and for the absence of the initiation codon in the Ensembl transcript. Therefore, ENST00000394900.7 should hybridize with oligonucleotide E17E18 and ENST00000529479.5 with INS50. GTEx data for these two transcripts (Table 2) indicated a 3.3-fold stronger expression of the latter in the heart, in relative agreement with the results of the northern assay (Figure 6a,b). Nonetheless, the same was observed in the GTEx database for the other tissues included in the northern assay, with 1.6–3.9-fold stronger expressions of the transcript encoding the X8-like isoform (Table 2).

An intriguing observation in the GTEx database was that the global expression of the *TKFC* gene in many tissues, including the small intestine, liver, and kidney (tissues showing a strong expression) was >90% attributable to 19 short transcripts unrelated to those coding for isoforms X6 or X8 (see https://gtexportal.org/home/gene/TKFC, accessed on 25 August 2024). Among these short transcripts, the high expression is particularly noticeable of ENST00000525366.5 (Table 2), a non-coding RNA that includes partly exons 14–18 of the *TKFC* gene model of Figure 2 which is theoretically able to hybridize with the probe E17E18.

## 4. Discussion

### 4.1. Possible Role of the Inactive TKFC Isoform X8

The short TKFC isoform X8 studied in this manuscript is inactive as triokinase and FMN cyclase. Therefore, it may not play a metabolic role in the fructose pathway or in the synthesis of cFMN. However, since the N domain of isoform X8 is, at least sequentially, like that of isoform X6, interaction with MDA5 and inhibition of MDA5-mediated innate immunity may still be possible. This should be investigated in future work.

In genetic studies, the absence of TKFC catalytic activities in biallelic mutants of isoform X6 has been related to several phenotypes including cataracts and multi system disease, developmental delay, liver dysfunction, microcytic anemia, fatal cardiomyopathy [24], hypotrichosis with loose anagen hairs [23], and an isolated, complex primary immunodeficiency [25]. One could speculate about the possible relation between any of those pathologies and the co-expression of the inactive TKFC isoform X8 with the active isoform X6. Since it is known that active TKFC is a homodimer of isoform X6, one wonders what would occur in the event of the formation of mixed dimers X6/X8. Would they show half the activity of X6 homodimers, or would they be inactive? In other words, X8 could show either incomplete dominance over X6 or act as a dominant negative isoform.

### 4.2. Comparison of the TKFC Transcripts Recorded in Databases with Those Detected in the Northern Blot

The length of the cloned sequence that contains the ORF coding for TKFC protein isoform X6 (DQ138290) is 1728 bp. This protein isoform is the enzymatic protein characterized as triokinase and FMN cyclase [1,2,28]. Figure 6a displays a major hybridization signal of about 2.2 kb, which is long enough to contain the complete coding sequence of isoform X6. Nevertheless, all the full-length RefSeq transcripts that theoretically code for isoform X6 are 4.6–6.0 kb long (Table 1), including the Ensembl transcript ENST00000394900.7. Therefore, although the major 2.2 kb RNA detected by hybridization to E17E18 must contain exons 17 and 18 spliced together by the removal of intron 17, one has to recall that the northern assay demonstrates hybridization to probes but does not prove the full-length character of the detected transcripts. The 2.2 kb RNA may represent artifactually shortened transcripts or *tkfc*-type transcripts not recorded in the databases. Also worthy of note is the absence of any northern signal around 0.9 kb (Figure 6a), which would correspond to the highly expressed Ensembl transcript ENST00000525366.5 according to the GTEX database (Table 2).

The cloned sequence that contains the ORF translating to TKFC protein isoform X8 (DQ344550) is 1784 bp long. Isoform X8 is the enzymatically inactive protein described in the current manuscript. Figure 6b shows major hybridization signals of about 4.3 kb and 1.6 kb (heart), 2.2 kb (liver), and 1.8 kb (placenta). All of them, except the 1.6 kb signal, can contain the complete coding sequence of isoform X8. However, only the 4.3 kb signal of the heart sample may correspond to one of the full-length RefSeq transcripts, namely, transcripts 5 or X14, which code for the very similar protein isoforms d and X8, respectively, or transcript 6, which codes for protein isoform e (Table 1, Figure 1). The remaining northern blot signals detected with the INS50 probe, either full-length transcripts or not, may represent artifactually shortened transcripts or *tkfc_ins56*-type transcripts not recorded in the databases. On the other hand, the 1.8 kb signal detected in the placenta is compatible with Ensembl transcript ENST00000529479.5, but this is unlikely to be full-length, as it lacks the initiation codon and any 5′-UTR. Therefore, this Ensembl transcript is probably part of a full-length one.

### 4.3. Comparison between tkfc-Type and tkfc_ins56-Type Splices of Intron 17 in Different Human Tissues

The distribution of *TKFC* transcripts in human tissues, detected by Northern blot with specific probes (Figure 6a,b), did not correlate with the global expression profile of the human *TKFC* gene analyzed by RNA-Seq in the same set of tissues (Figure 6c and GTEx database). The difference between both approaches is better appreciated in terms of the relative levels of liver:intestine *TKFC* expression. While the liver sample gave a much stronger E17E18 (or INS50) signal than the intestine, the latter tissue displayed the largest frequency of *TKFC*-derived tags in the RNASeq profile. In addition, comparisons of the heart with other tissues (e.g., brain, skeletal muscle, colon, thymus, spleen, lung) led to a less contrasted but still clear lack of correlation between Figure 6b,c, since the INS50 signals of the heart were stronger than those in the other tissues, while in the RNASeq profile, all of them displayed similar or higher frequencies of *TKFC* tags than the heart. The major reason for the lack of correlation between the northern assay and global expression data may be that global *TKFC* expression, assayed by RNA-Seq, reflects a very high proportion sequence tags derived from short transcripts unrelated to those coding for isoforms X6 or X8.

It must be remarked that the Northern blot of Figure 6a,b and the data available in GTEx (Table 2) for the relative levels of *tkfc*-type and *tkfc_ins56*-type splices in different tissues do not correlate with each other. This can be attributed to several factors: (i) differences in the sources of RNA: poly(A)-RNA from one donor in the northern assay and poly(A)-selected RNA from many donors in the RNA-Seq assay of the GTEx database; (ii) uncertainty in the estimation of transcript levels due to difficulties in assigning the RNA-Seq reads to a specific transcript; and (iii) the complexity of the transcript set derived from the *TKFC* gene, namely, 21 Ensemble transcripts in the GTEx database (https://gtexportal.org/home/gene/TKFC, accessed on 25 August 2024) and 22 recorded in GenBank (Figure 1), with few coincidences between both databases.

Altogether, expression data for the *TKFC* gene seem to imply that, at least in the small intestine, but possibly also in other tissues, the predominant *TKFC* spliced transcripts did not hybridize with E17E18 (nor with INS50) and therefore had not undergone *tkfc*- or *tkfc_ins56*-type splicing of intron-17. An exception to this is the high expression of the short ENST00000525366.5 transcript detected by RNA-Seq (Table 2) but not by northern assay (Figure 6a). Anyhow, the results support the occurrence of potentially relevant transcript variants of the *TKFC* gene differentially expressed in human tissues. Further experimental research with more quantitative techniques may give a deeper understanding of this matter.

## 5. Conclusions

In conclusion: (i) a human brain transcript variant, that contains an alternative 3′-splice of *TKFC* intron 17, was cloned, sequenced and expressed; (ii) with respect to transcripts coding for active triokinase and FMN cyclase, the new transcript contains a 56-nt insertion between exons 17 and 18 (*tkfc_ins56*-type transcripts), with an in-frame stop codon; (iii) it encodes a shortened and enzymatically inactive protein identified as TKFC isoform X8, which lacks 50 C-terminal amino acids encoded by *TKFC* exon 18 and contains instead 9 amino acids encoded by intron 17; (iii) the lack of triokinase activity of isoform X18 was related to the absence of the essential residue Arg543; (iv) the results depict two pathways for *TKFC* intron 17 splicing: *tkfc*-type splicing, which leads to the direct junction of exons 17 and 18, and *tkfc_ins56*-type splicing, which leads to an insertion of 56 nt between exons 17 and 18; (v) in a northern assay, in the liver, the *tkfc*-type splice is strongly predominant, while in the heart, the *tkfc_ins56*-type splicing is so; (vi) in the small intestine, *TKFC* transcripts of *tkfc*- or *tkfc_ins56*-type were not detected, in contrast to the strong expression of the *TKFC* gene determined by RNAseq, what supports that a different pathway of splicing is predominant in the intestine; (vii) northern blot data do not correlate well with the levels of transcripts quantified by RNA-Seq as shown in the GTEx database, what can be explained by technical differences between both approaches; (viii) altogether, this study supports the occurrence of potentially relevant variants of human *TKFC* transcripts differentially expressed in human tissues.

## Figures and Tables

**Table 1:** *TKFC* full-length transcripts (splice variants) and their encoded protein isoforms. The exon structures of the transcript variants are depicted in Figure 1.

*TKFC* Transcript Variant ^1^				TKFC Protein Isoform ^2^		
Name	Accession no.	Length (nt)	Intron 17 Splice Type	Name	Accession no.	Same as
1	NM_001351977.2	2252		a	NP_001338906.1	
2	NM_015533.4	4673	*tkfc* ^3^	b	NP_056348.2	T185A-X6
3	NM_001351976.2	4755	*tkfc*	b	NP_001338905.1	T185A-X6
4	NM_001351978.2	2353		c	NP_001338907.1	T185A-X7
5	NM_001351979.2	4246	*tkfc_ins56* ^4^	d	NP_001338908.1	T185A-X8
6	NM_001351980.2	4344	*tkfc_ins56*	e	NP_001338909.1	
X1	XM_054368358.1	3841		X1	XP_054224333.1	
X2	XM_054368359.1	5988	*tkfc*	X2	XP_054224334.1	
X3	XM_054368360.1	3668		X3	XP_054224335.1	
X4	XM_054368361.1	3823		X4	XP_054224336.1	
X5	XM_054368363.1	2526		X4	XP_054224338.1	
X6	XM_054368364.1	2628		X4	XP_054224339.1	
X7	XM_054368365.1	5561	*tkfc_ins56*	X5	XP_054224340.1	
X8	XM_054368366.1	5970	*tkfc*	X6	XP_054224341.1	A185T-b (ABA10576.1) ^5^
X9	XM_054368368.1	5001	*tkfc*	X6	XP_054224343.1	A185T-b (ABA10576.1) ^5^
X10	XM_054368369.1	3650		X7	XP_054224344.1	A185T-c
X11	XM_054368370.1	2455		X7	XP_054224345.1	A185T-c
X12	XM_054368371.1	5543	*tkfc_ins56*	X8	XP_054224346.1	A185T-d (ABC70184.1) ^6^
X13	XM_054368372.1	5317	*tkfc_ins56*	X8	XP_054224347.1	A185T-d (ABC70184.1) ^6^
X14	XM_054368373.1	4348	*tkfc_ins56*	X8	XP_054224348.1	A185T-d (ABC70184.1) ^6^
X15	XM_054368362.1	3597		X4	XP_054224337.1	
X16	XM_054368367.1	5744	*tkfc*	X6	XP_054224342.1	A185T-b (ABA10576.1) ^5^

^1^ Transcript variants 1–6, with NM accessions, are RefSeq curated sequences derived mainly from mRNA submissions to GenBank; transcript variants X1–X16, with XM accessions, are predictions derived from the sequence of the T2T-CHM13v2.0 human genome. ^2^ Protein isoforms are the theoretical translations of the transcript variants. ^3^ *tkfc* denotes the constitutive splicing of intron 17 occurring, e.g., in transcripts coding for TKFC. ^4^ *tkfc_ins56* denotes the alternative splicing of intron 17 occurring by through a weak 3′-splicing site. ^5^ ABA10576.1 is the accession number of the translation of a cDNA sequence (accession no. DQ138290.1) earlier cloned and expressed as active triokinase and FMN cyclase (TKFC protein isoform X6) [1,2,28]. ^6^ ABC70184.1 is the accession number of the translation of a cDNA sequence (DQ344550.1) described and used in the current manuscript to express the catalytically inactive protein isoform X8.

**Table 2:** Expression data of Ensembl *TKFC* transcripts theoretically hybridizing with the probes INS50 or E17E18 according to the GTEx database (https://gtexportal.org/home/gene/TKFC; accessed on 25 August 2024).

Transcript ID	ENST00000529479.5	ENST00000394900.7	ENST00000525366.5
Length	1781 nt	4678 nt	916 nt
Hybridize with probe	INS50	E17E18	E17E18
Protein isoform encoded	T185A+F437S-X8 ^1^	T185A-X6	None
	Transcripts per million		
Brain (mean of 11 locations)	2.27	1.40	6.93
Heart (mean of 2 locations)	1.72	0.52	3.21
Skeletal Muscle	2.34	0.68	2.99
Colon	4.73	1.99	8.79
Thymus	not available	not available	not available
Spleen	6.22	1.84	13.50
Kidney (mean of 2 locations)	8.37	2.76	21.15
Liver	17.40	4.47	47.80
Small Intestine	26.00	6.93	50.60
Placenta	not available	not available	not available
Lung	4.73	1.97	10.70

^1^ Assuming the presence of an initiation codon before the Ensembl sequence.

## Data Availability

The original contributions presented in the study are included in the article/Supplementary Material, further inquiries can be directed to the corresponding authors.

## Supplementary Materials

The following supporting information can be downloaded at: https://www.mdpi.com/article/10.3390/biom14101288/s1, Original images of Figure 3a,b and Figure 6a,b can be found in Supplementary Materials.
