# Supplementary figures and images for: Alternative Splicing of the Last TKFC Intron Yields Transcripts Differentially Expressed in Human Tissues That Code In Vitro for a Protein Devoid of Triokinase and FMN Cyclase Activity
Source: Biomolecules. 2024 Oct 12;14(10):1288. doi: 10.3390/biom14101288 (PMC11506722; doi:10.3390/biom14101288)

MW

Figure 3a

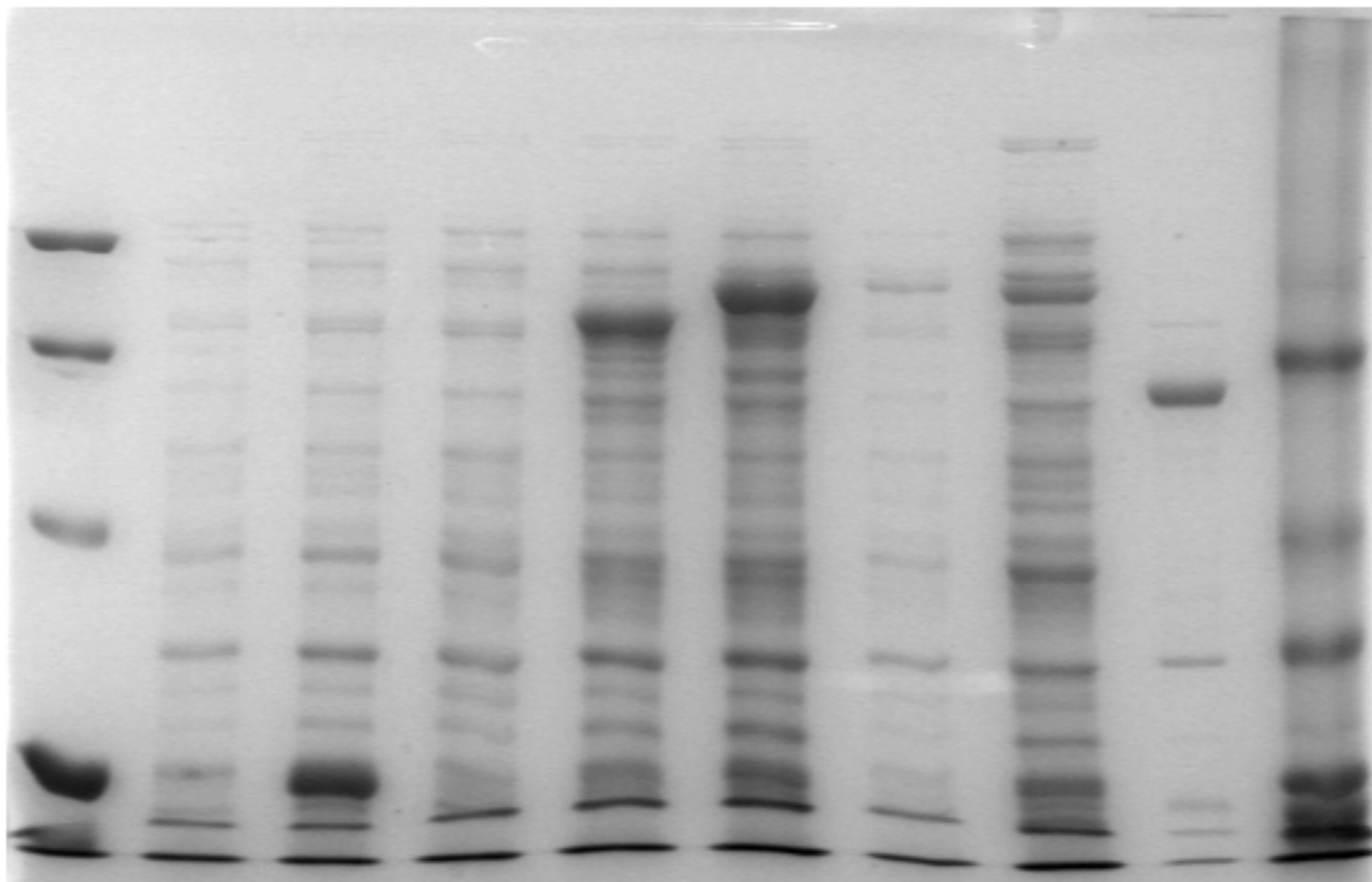

Supplement: Supplementary file 1 [file biomolecules-14-01288-s001.zip › Figure 3a.pdf]

MW

Figure 3b

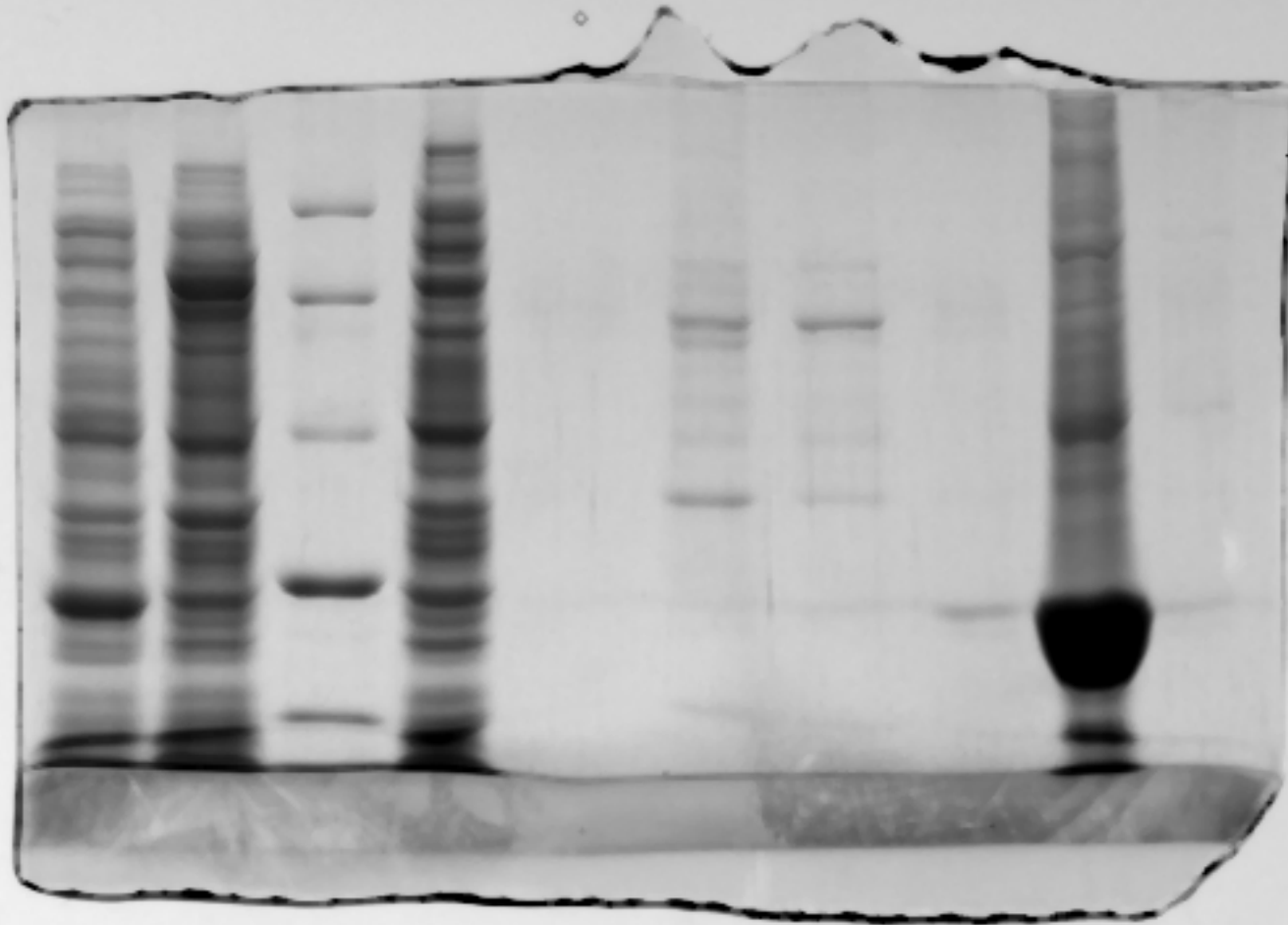

Supplement: Supplementary file 1 [file biomolecules-14-01288-s001.zip › Figure 3b.pdf]

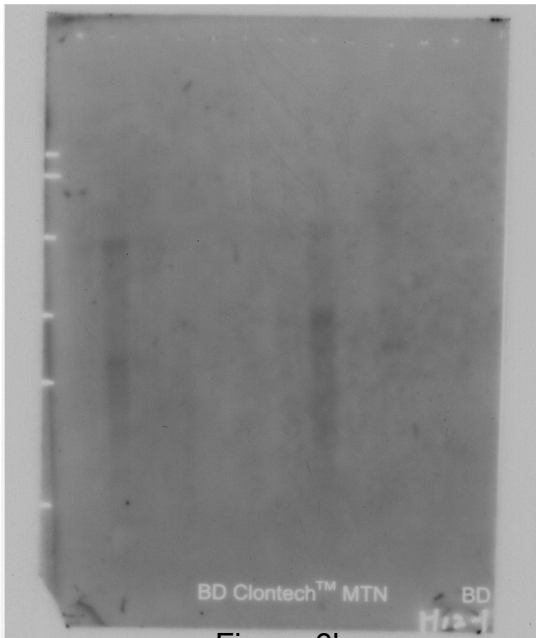

Figure 6b

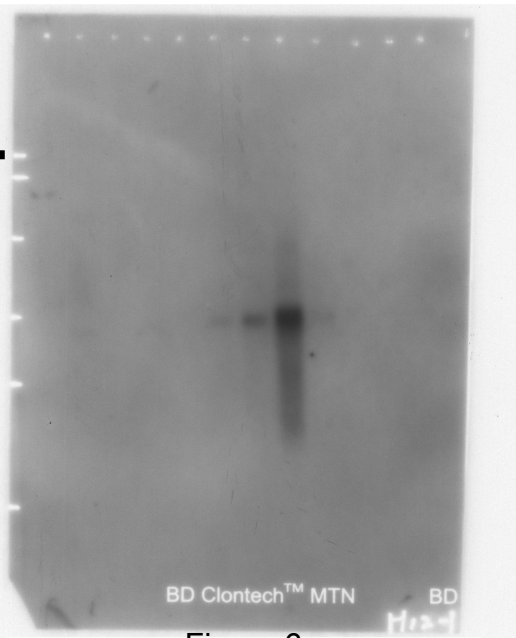

Figure 6a

Supplement: Supplementary file 1 [file biomolecules-14-01288-s001.zip › Figure 6a,b.pdf]
